# Compliance of Primary Care Providers With Gout Treatment Recommendations—Lessons to Learn: Results of a Nationwide Survey

**DOI:** 10.3389/fmed.2020.00244

**Published:** 2020-06-02

**Authors:** Judith Sautner, Thomas Sautner

**Affiliations:** ^1^Second Medical Department, Korneuburg-Stockerau Hospital, Lower Austrian Centre for Rheumatology, Stockerau, Austria; ^2^Medical Directorate, Hospital of St. John of God, Vienna, Austria

**Keywords:** primary health care, compliance, gout management recommendations, gout knowledge questionnaire, treatment patterns, gout flare, serum uric acid target, urate lowering therapy (ULT)

## Abstract

**Objectives:** Gout is generally managed in the primary health care sector. Compliance of primary care physicians with gout management recommendations has been shown to be insufficient in the past. The primary aims of this study were to assess primary care providers' knowledge regarding gout and to determine if their treatment decisions are influenced by recommendations. Facing considerable variations in postgraduate training options in Austria, we secondly looked for possible knowledge differences between urban and rural areas and eastern and western parts of Austria.

**Methods:** We conducted a survey among 343 primary care physicians in Austria, using a questionnaire consisting of 10 questions on acute, intercritical and general gout management. Gout treatment recommendations served as the therapeutic gold standard.

**Results:** Of the 343 physicians surveyed, 336 completed the questionnaire, leading to a very high return rate of 98%. 289 (86%) physicians were aware of the agreed-upon SUA target (<6 mg/dl). 323 (96.1%) reported change of therapy in case of missing this target. 112 (33.3%) physicians voted for long term ULT. No geographical differences in knowledge regarding gout or its management were revealed, except that colchicine was rated as being a safe medication significantly more often in rural areas (*p* = 0.035) and in western Austria (*p* = 0.014).

**Conclusion:** As opposed to former studies, gout knowledge among primary care physicians has improved - however, treatment patterns are still not fully concordant with gout recommendations. Our findings may help to better tailor future postgraduate training to improve primary care physicians' education in gout.

## Introduction

Gout, with a prevalence of ~2%, is the leading cause of inflammatory arthritis within developed countries. Left untreated, gout has the potential to advance into a chronic stage, including joint destruction and associated problems (1). Globally, especially in countries with a high socio-demographic index, a growing prevalence can be observed (2). An association between gout and various internal diseases is generally acknowledged (3–6). Gout is an independent predictor of cardiovascular morbidity and mortality, although causal pathways have not yet been fully elucidated (7–9). Moreover, an extended therapeutic armamentarium with new urate-lowering therapies (ULT) has also elevated awareness about gout. Thus, a number of gout recommendations have been established by various rheumatologic societies (10–15). Gout is mainly managed in the primary care sector. Despite the multitude of recommendations available, the standard of gout management is not yet optimal and lags behind the research (16–18). Therefore, research, especially in primary care, is proposed (10).

The primary aims of the present study were to assess primary care physicians' knowledge regarding gout and their adherence to current treatment recommendations as the gold standard. Thus, therapeutic and general gout management was evaluated across Austria. As continuous advanced medical training is compulsory in Austria, approved postgraduate training is offered by the Austrian academy of physicians (Akademie der Ärzte), the educational branch of the Austrian medical association. According to the 2018 report of the Austrian academy of physicians, there is a regional variability in trainings offered. Trainings in internal medicine and rheumatology were offered in a considerable range from 720 (Vienna) to 98 (Burgenland)^1^. In relation to the number of primary care physicians per province, trainings offered per physician range from 0.54 (Upper Austria) to 0.95 (Vienna)^2^. Therefore, as a secondary aim, we also looked for differences in gout-related knowledge between urban and rural areas and between eastern and western parts of the country, respectively.

## Materials and Methods

### Participants

All physicians practicing in Austria are obliged to be members of the Austrian Medical Association (AMA) and are registered in an official list. This list served as the data source for the selection of the participants. Comparable to similar surveys, we aimed at a sample size of 5–10% of all Austrian primary care physicians. From January till March 2019, fourteen pharmaceutical representatives were instructed to randomly pick twenty-five physicians each from the AMA-list according to their catchment areas. They distributed the questionnaires directly to the physicians' offices, along with prepaid return envelopes. A financial incentive of €50 was offered for completing the questionnaire.

### Questionnaire

We developed an anonymous, self-administered questionnaire, consisting of ten questions to assess clinical knowledge and beliefs and to investigate therapeutic patterns for acute and intercritical gout as well as general gout management (see Table 1). The questions were selected and adapted from a previous questionnaire of 17 questions, successfully tested and used in a survey among Austrian rheumatologists in 2013 (19). Again, the content of current gout treatment recommendations (EULAR recommendations, the evidence based S2e guidelines of the German Society of Rheumatology and the International 3e- as well as the Austrian 3e-recommendations for the management of gout) served as the gold standard (10, 12, 14, 15). These recommendations are concordant regarding questions 1–6, 9 and 10. Question 7 regarding the duration of ULT is addressed in the EULAR and International 3e- and Austrian 3e-recommendations. Question 8 is addressed only in the German S2e guideline.

**Table 1:** Questionnaire, MTP I = metatarsophalangeal joint I, OD = once daily (correct answers are highlighted in bold).

1. Medication of choice for the management of an acute gout flare is: (multiple answers allowed)
a. **NSAIDs**
b. **Steroids**
c. **Combination of NSAIDs and corticosteroids**
d. **Colchicine**
2. Colchicine is a safe and efficient medication with a manageable amount of side effects
a. **Yes**
b. **Yes, with limitations**
c. No, I never or hardly ever use it
d. I don't have any experience with colchicine.
3. I am starting ULT in case of (multiple answers allowed)
a. **Recurrent gout flares**
b. **Typically affected joints (e.g., MTP I)**
c. Asymptomatic hyperuricemia
d. **Tophi**
4. When applying ULT, I am aiming at a serum uric acid level of:
a. <10 mg/dl
b. <8 mg/dl
c. **<6 mg/dl**
d. <3 mg/dl
5. If the serum uric acid target cannot be achieved by allopurinol (300mg OD), (multiple answers allowed)
a. **I increase the dosage of allopurinol**
b. **I switch from allopurinol to febuxostat**
c. **I add the uricosuric lesinurad to allopurinol**
d. I wait and see
6. I would switch from allopurinol to febuxostat due to: (multiple answers allowed)
a. **Insufficient ULT by allopurinol**
b. **Incompatibility or allergic reaction to allopurinol**
c. At patient's request
7. I prescribe ULT (multiple answers allowed)
a. For 6 months following the onset of acute gout
b. **For 5 years**
c. **Life long**
8. I measure the serum uric acid level (multiple answers allowed)
a. During an acute gout flare
b. **2 weeks after a flare at the earliest**
c. **Regularly every 3 months under current ULT**
d. At rare intervals, not even every 12 months
9. The modification of diet and lifestyle in case of gout/hyperuricemia is (multiple answers allowed)
a. **Important and should be discussed with every patient.**
b. Maybe suitable for some patients but is not part of my general recommendations for gout.
c. **Can be explained much easier using informative visual aids**
d. This issue is not part of my medical consultation.
10. In patients with hyperuricemia, the cardiovascular risk
a. is as high as in the general population
**b. is increased**
c. remains to be determined

The comprehensibility of the questionnaire was tested by interviews with two primary care physicians. The questionnaire was intentionally kept short and easy to complete in order to achieve a high return rate. Some of the questions had a multiple-choice response option. The questionnaire included a short explanation how to complete it. It was indicated to answer the questions according to the participants' knowledge of current national or international guidelines. All items with multiple answers allowed were indicated. Given several recommendations as gold standard, to gather the whole body of knowledge, the authors agreed to mark multiple-choice answers, as follows: full accordance if all answers were ticked in accordance with recommendations and as partial accordance if some answers conforming with recommendations were ticked. If both conforming and nonconforming answers were given, the answer was marked as partly conflicting and, if no concordance with recommendations at all was given, it was marked as fully conflicting. Concomitantly, basic demographic data for participants was collected, along with data on location and catchment area of physicians' practices in order to differentiate between urban and rural areas. Completion time for the questionnaire was tested beforehand on five primary care physicians and took <5 min. No patients were involved in the study and no patient data was obtained in the questionnaire. In accordance with the national legislation, written informed consent for participation was not required for this study. Because the study only involved voluntary participation and did not include any further intervention, a retrospective waiver of ethics approval was sufficient and has been obtained from the Lower Austrian Ethics committee.

### Analysis

For initial analyses, descriptive statistics were used. For continuous variables, we calculated the mean as well as the standard deviation (SD). For categorical variables, proportions were calculated. We tested categorical data with the chi-square test. *P*-values < 0.05 were considered statistically significant. For unequal distributions, weighted contingency tables have been calculated.

## Results

### Respondents

Out of 343 physicians approached, 339 responded. 336 questionnaires were complete which represents a return rate of 98%. We were able to gather participants from eight of nine Austrian provinces (for demographics and regional distribution of participating physicians see Table 2). The proportions of participants per province in our sample were similar to the proportions of overall primary care physicians per province in six of the comprised provinces. The rate of participating physicians in Upper Austria only reached 12% of the pursued rate whereas in Carinthia it exceeded the pursued rate 2.6 fold. The overall participation rate amounted to 8.5% (see Table 3).

**Table 2:** Demographics and regional distribution of participating physicians.

**Age (mean ± SD)**	**50.3 (±9.3)**	
Male	171 (51%)	
Female	165 (49%)	
Private practice catchment area (population of municipality)		
<5,000 residents	85 (26.2%)	
5,000–10.000 residents	99 (30.6%)	
>10.000 residents	140 (43.2%)	
	**Study sample**	**Overall Austria**
Private practices in larger cities		
(Vienna, Graz, Linz, Innsbruck, Salzburg)	97 (28.6%)	1065 (26.8%)
Private practices in smaller cities and rural areas	242 (71.4%)	2909 (73.2%)
Eastern Austria		
(Vienna, Burgenland, Styria, Lower Austria)	232 (69%)	2278 (57.3%)
Western Austria		
(Upper Austria, Carinthia, Salzburg, Tyrol)	104 (31%)	1696 (42.7%)

**Table 3:** Number of participating primary care physicians per province (PP), participation rate in relation to the sample size (PR), total number of primary care physicians per Austrian province (TP), relative number of primary care physicians per Austrian province (RPP).

**Province**	**PP**	**PR (%)**	**TP**	**RPP (%)**
Vienna	69	20.5	759	19.1
Burgenland	11	3.3	142	3.6
Styria	71	21.1	593	15.0
Lower Austria	81	24.1	784	19.7
Upper Austria	7	2.1	716	18.1
Carinthia	53	15.7	244	6.1
Salzburg	19	5.6	261	6.6
Tyrol	25	7.4	318	8.0
Vorarlberg	0	0	157	3.9
Austria (total)	336	100	3974	100

### Management of Acute Gout Flares

For the management of acute gout flares, NSAIDs combined with corticosteroids are the medication of choice (107/31.8%), followed by NSAID monotherapy or combined with colchicine. 81 physicians (24.1%) ticked all available options, thereby giving no preference.

Colchicine is regarded as a safe medication by 241 (71.7%) physicians, with 61 (64%) in urban areas vs. 180 (75%) in rural areas, which is significantly different (*p* = 0.035). Likewise, 157 (68%) physicians in eastern Austria consider colchicine a safe medication vs. 84 (81%) in western Austria, which is also significantly different (*p* = 0.014) (see Table 4).

**Table 4:** Management of acute gout, *n* = number of physicians (%).

	**Urban**	**Rural**	***p***	**East**	**West**	***p***
**Q1: Medication of choice for acute gout**						
NSAID	22 (23)	41 (17)		52 (22)	11 (11)	
Steroid	0	4 (2)		2 (1)	2 (2)	
Colchicine	5 (5)	9 (4)		8 (3)	6 (6)	
NSAID+steroid	29 (30)	78 (32)		69 (30)	38 (36)	
NSAID+colchicine	22 (23)	41 (17)		48 (21)	15 (14)	
Steroid+colchicine	0	4 (2)		3 (1)	1 (1)	
No. preference	18 (19)	63 (26)		50 (22)	31 (30)	
Total	96	240	0.26	232	104	0.07
**Q2: Colchicine is**						
Safe	61 (64)	180 (75)		157 (68)	84 (81)	
Not safe	35 (36)	60 (25)		75 (32)	20 (19)	
Total	96	240	0.035	232	104	0.014
Safe = a,b						
Not safe = c,d						

### Urate Lowering Therapy

The question on the use of ULT, representing the management of intercritical recurrent gout, is answered correctly by less than a quarter of physicians (23% in rural areas, 18% in urban areas). 213 (63.4%) responses are at least partly in line with the applied current recommendations.

Concerning the serum uric acid (SUA) target, 289 (86%) answered correctly in accordance with the generally recommended SUA target of <6 mg/dl. There was no observable statistical difference between urban and rural areas nor between eastern and western Austria (*p* = 0.08).

The vast majority of physicians (323/96.1%) would actively change their therapeutic regime if the SUA target was not yet reached. Among these, 271 (80.7%) would switch medications from allopurinol to febuxostat. 12 (3.6%) would only increase the allopurinol dose. 2.4% would wait and see.

210 (62.5%) of physicians stated that they would switch from allopurinol to febuxostat if the former was no sufficient ULT or if patients would experience an allergy or incompatibility to allopurinol. 27 (8%) explicitly addressed an incompatibility or allergic reaction to allopurinol as the main reason for a switch.

A third of physicians chose long term ULT. Incorrect answers were obtained from 165 (49.1%) responders (see Table 5).

**Table 5:** Management of recurrent gout, ULT = urate lowering therapy, *n* = number of physicians (%).

	**Urban**	**Rural**	***p***	**East**	**West**	***p***
**Q3: Start of ULT (according to current treatment recommendations)**						
Full accordance	17 (18)	54 (23)		49 (21)	22 (21)	
Partial accordance	39 (41)	103 (43)		92 (40)	50 (48)	
Partly conflicting	24 (25)	55 (23)		62 (27)	17 (16)	
Fully conflicting	16 (17)	28 (12)		29 (13)	15 (14)	
Total	96	240	0.52	232	104	0.19
Full accordance = a+b+d						
Partial accordance = a,b,d,a+b,a+d,b+d						
Partly conflicting = any combination containing c						
Fully conflicting = c						
**Q4: Serum uric acid target level**						
<10 mg/dl	1 (1)	3 (1)		1 (0.5)	3 (3)	
<8 mg/dl	14 (15)	28 (12)		27 (11.5)	15 (14)	
<6 mg/dl	81 (84)	208 (87)		204 (88)	85 (82)	
<3 mg/dl	0 (0)	1 (0.4)		0	1 (1)	
Total	96	240	0.82	232	104	0.08
**Q5: Measures to take in case serum uric acid target level is not reached**						
Full accordance	94 (98)	229 (95)		224 (96.5)	99 (95)	
Partly conflicting	0	5 (2)		2 (1)	3 (3)	
Fully conflicting	2 (2)	6 (3)		6 (2.5)	2 (2)	
Total	96	240	0.35	232	104	0.35
Full accordance = a,a+b,a+b+c,b,b+c,c						
Partly conflicting = b+c+d,b+d						
Fully conflicting = d						
**Q6: Reasons for switching from allopurinol to febuxostat**						
Insuff.ULT by allopurinol	18 (19)	67 (28)		53 (23)	32 (31)	
Allergy to allopurinol	11 (11)	16 (7)		19 (8)	8 (8)	
Both	62 (65)	148 (62)		150 (65)	60 (58)	
Both+pat.request	5 (5)	9 (3)		10 (4)	4 (4)	
Total	96	240	0.19	232	104	0.5
**Q7: Duration of ULT according to recommendations**						
Full accordance	28 (29)	84 (35)		76 (33)	36 (35)	
Partly conflicting	22 (23)	37 (15)		39 (17)	20 (19)	
Fully conflicting	46 (48)	119 (50)		117 (50)	48 (46)	
Total	96	240	0.23	232	104	0.75
Full accordance = b,c,b+c						
Partly conflicting = a+b,a+b+c,a+c						
Fully conflicting = a						

### Measurement of Serum Uric Acid

The correct moment in time for measurement of SUA was given by 53.6% of the responding physicians. Incorrect answers were obtained from only 3%.

### Diet and Lifestyle

93.8% of physicians correctly acknowledged diet and lifestyle as important factors that have to be addressed in patients with gout. An elevated cardiovascular risk of gout patients was acknowledged by 92.3% (see Table 6).

**Table 6:** General management of gout, including lifestyle, *n* = number of physicians (%).

	**Urban**	**Rural**	***p***	**East**	**West**	***p***
**Q8: Moment in time for measurement of serum uric acid level**						
Full accordance	48 (50)	132 (55)		122 (53)	58 (56)	
Partial accordance	17 (18)	54 (22.5)		48 (21)	23 (22)	
Partly conflicting	29 (30)	46 (19)		56 (24)	19 (18)	
Fully conflicting	2 (2)	8 (3.5)		6 (3)	4 (4)	
Total	96	240	0.16	232	104	0.64
Full accordance = b+c						
Partial accordance = b,c,b+c+d						
Partly conflicting = a+b+c,a+c,a+c+d,b+d						
Fully conflicting = a,a+d,d						
**Q9: Influence of diet and lifestyle on gout**						
Full accordance	89 (93)	226 (94)		217 (94)	98 (94)	
Partly conflicting	7 (7)	14 (6)		15 (6)	6 (6)	
Total	96	240	0.62	232	104	0.81
Full accordance = a,a+b,a+b+c,a+c,c						
Partly conflicting = a+b+c+d,a+c+d,a+d, b,b+c						
**Q10: Cardiovascular risk in gout patients**						
Full accordance	89 (93)	221 (92)		213 (92)	97 (93)	
Fully conflicting	7 (7)	19 (8)		19 (8)	7 (7)	
Total	96	240	0.84	232	104	0.64
Full accordance = b						
Fully conflicting = a,c						

## Discussion

In a nationwide survey on knowledge and treatment practices for gout, we gathered data from primary care physicians and were able to achieve a return rate of 98%. Our sample represents 8.5% of the primary care physicians of Austria, a rate which is comparable to a similar survey in the US (20). Our study population represents the relative distribution of Austrian private practices with respect to urban and non-urban regions as well as the relative distribution for the majority of the Austrian provinces. As a limitation, two provinces are underrepresented whereas one is overrepresented. However, an additional weighted analysis of our data revealed no difference of results in comparison to the not weighted analysis.

Austrian primary care physicians are aware of current treatment recommendations for gout, to a greater extent than comparable surveys have shown (16, 20). While the pursued SUA target and the change of therapeutic regimes in case of not reaching it are well known, we observed deficits concerning the correct moment in time for SUA measurement, the correct start of ULT and its duration. In contrast to earlier investigations, the importance of addressing diet and lifestyle with gout patients is widely acknowledged (20). Despite regional variations in postgraduate training, no major differences in compliance with treatment recommendations between different regions of Austria could be observed.

### Comparison With Former Surveys

Gout is largely managed by primary care physicians. It is therefore eminently important to ensure familiarity with rheumatologic recommendations for the management of gout. A few surveys on the compliance of clinicians' work with current guidelines have been conducted, using different designs and approaches - some among rheumatologists, others among primary care providers (16, 19–25). Our survey is—to our knowledge—the first one to compare urban and rural areas and different geographical regions, when assessing awareness of gout management recommendations and treatment patterns in the primary care setting. In contrast to similar studies in France and the US, we were able to achieve a considerably higher percentage of participating physicians. Also, the return rate in our survey was significantly higher (16, 26). This may have been positively influenced by two factors: personal delivery of questionnaires by traveling pharmaceutical staff on the one hand and offering a financial incentive for the completion of the questionnaire on the other. The latter had also been applied in one of the above-mentioned surveys (20). The inclusion of monetary incentives to partially compensate physicians for time spent completing a survey and also to show appreciation for their efforts is supported by the literature (26). The selection of participating physicians was representative, as their demographics perfectly match those of all Austrian primary care physicians (mean age 53.3 years, 48% female)^3^ (Table 2).

### Management of Acute Gout

Regarding therapy for acute gout flares, NSAIDs in combination with corticosteroids are the preferred therapeutic strategy in Austria. This is in line with literature suggestions for gout. NSAIDs are also the preferred treatment for musculoskeletal pain among primary care physicians (27, 28). Colchicine is part of all recommendations for acute gout management as well as for the prophylaxis of recurrent flares. In the survey conducted among Austrian rheumatologists in 2013, only 17% of the responders considered colchicine as a safe medication (19). In the present survey, this percentage was higher and especially pronounced in rural areas, reaching statistical significance. Also, significantly more physicians in western Austria consider colchicine a safe medication as compared to eastern Austria. This observation is difficult to interpret, given the fact, that less postgraduate education is offered outside large cities and in western Austria^1^.

### Management of Recurrent Gout

Apart from the correct answers regarding different indications for initiating ULT, more than one third of all participants also voted for starting ULT in the case of asymptomatic hyperuricemia, either per se or in combination with other indications (multiple answers allowed). Whether or not the participating primary care physicians were influenced by reports on positive effects of ULT in terms of internal comorbidities e.g., coronary artery disease, cannot be answered (29). Notably, we and others observed that the majority of rheumatologists comply with gout recommendations, not prescribing ULT in case of asymptomatic hyperuricemia (19, 22), whereas more than half of the interviewed specialists in internal medicine would treat asymptomatic hyperuricemia (22).

All gout therapy recommendations unanimously agree on the lifelong maintenance of the SUA target, once achieved. Knowledge about the SUA target <6 mg/dl in the treat to target approach in gout tends to be higher among the study participants than in former surveys (20, 22). Knowledge about possible risks of a SUA level below 3 mg/dl is as good (10). In summary, awareness of appropriate SUA target levels appears to be sufficient in the primary care sector. In case of not reaching the SUA target, the vast majority of physicians would modify their therapeutic strategy, which is also in line with the recommendations. Most physicians voted for a switch to febuxostat. Fewer would increase the allopurinol dose, which underscores earlier findings on allopurinol prescription practice (30–32).

Remarkably, we observed considerable differences in the prescription duration of ULT. Though the underlying recommendations agree upon the maintenance of a lifelong treatment, only a third of physicians voted for long term therapy. A possible explanation for the high percentage of answers favoring six months of ULT following an acute flare, could be confusion with the recommended duration of a flare prophylaxis. However, even in surveys among rheumatologists, long term maintenance of the SUA target once achieved, was identified as one of the main problems in gout management (25).

### General Gout Management

Only half of the physicians responded correctly with respect to the moment in time for taking SUA measurement. For the treat to target approach in gout, measurement of uric acid is part of most recommendations, while no exact moment in time for taking SUA measurement is explicitly mentioned (10–15, 33, 34). As already pointed out by others, the clear definition of time intervals to measure SUA is mandatory but still lacking (11).

As opposed to the limited knowledge on diagnostic and therapeutic measures, a huge majority of physicians was well informed about the increased cardiovascular risk of patients with gout and about the necessity and benefits of addressing diet and lifestyle with gout patients (35). Knowledge of primary care providers on general aspects and the epidemiologic background of gout has considerably improved in recent years (20).

It has been shown that a more intense care of gout patients leads to a better outcome with significantly more patients achieving the SUA target experiencing significantly fewer gout attacks (36). That underscores the importance of thorough and comprehensive management of gout in the primary health care setting. Primary care physicians consent to the benefits of a close collaboration with rheumatologists, who are also assigned to deliver and improve ongoing rheumatologic training for the primary care sector (37).

A possible shortcoming of the study is that we did not ask for the individual amount of recent postgraduate training in gout. However, having given the situation in Austria, that postgraduate training is compulsory for all physicians, we chose to take the number of trainings per province, offered by the official agency (Österreichische Akademie der Ärzte) as a marker of available knowledge^1^. Moreover, a discrepancy between reported knowledge and treatment patterns in daily clinical practice cannot be ruled out (22). In order to create a compact questionnaire, we did neither ask for the inclusion of patient data nor for details concerning co-medications. However, this lean approach led to the highest response rate in a survey on gout so far.

## Conclusion

In comparison to former findings, we observed a progress in knowledge regarding gout among primary care physicians. The participants show a high awareness of the pursued SUA target and tend to adjust their therapy when the SUA target is not achieved. The importance of addressing diet and lifestyle with gout patients as well as the increased cardiovascular risk of these patients is well known. However, there is still undeniable need for improvement regarding the issues of ULT and SUA measurement. Our findings may help to better tailor future postgraduate transfer of knowledge regarding gout for the primary care sector. A modified educational content could close the knowledge gaps revealed to improve the care of patients with gout.

## Data Availability Statement

The raw data supporting the conclusions of this article will be made available by the authors, without undue reservation.

## Ethics Statement

Ethical review and approval was not required for the study on human participants in accordance with the local legislation and institutional requirements. Written informed consent for participation was not required for this study in accordance with the national legislation and the institutional requirements.

## Author Contributions

JS: conception, design, acquisition of data and manuscript preparation. TS: statistical analysis and review of the manuscript.

## Conflict of Interest

The authors declare that the research was conducted in the absence of any commercial or financial relationships that could be construed as a potential conflict of interest.

## Footnotes

^1^Source: Ö*sterreichische Akademie der Ärzte GmbH*: https://www.arztakademie.at/fileadmin/template/main/DFP_FBBerichte/2019_AErztlicher_Fortbildungsbericht.pdf (personal communication with the Austrian academy of physicians).

^2^Source: Hauptverband der Österreichischen Sozialversicherungsträger, Austrian National Insurance, report on primary care physicians, 31.12.2015, page 8: https://www.sozialversicherung.at/cdscontent/load?contentid=10008.714996&version=1509372519

^3^Source: Austrian Medical Association, report on physicians' statistics, 31.12.2018, https://www.aerztekammer.at/documents/261766/124736/OeAeK+Aerztestatistik_2018.pdf/a7504601-fae6-938d-368d-1dc224bb8118

## Abbreviations

SUA: serum uric acid
ULT: urate lowering therapy
NSAID: non steroidal antirheumatic drug.
